# CkREV regulates xylem vessel development in *Caragana korshinskii* in response to drought

**DOI:** 10.3389/fpls.2022.982853

**Published:** 2022-08-25

**Authors:** Jiayang Li, Lifang Xie, Jiejie Ren, Tianxin Zhang, Jinhao Cui, Zhulatai Bao, Wenfei Zhou, Juan Bai, Chunmei Gong

**Affiliations:** ^1^College of Horticulture, Northwest A&F University, Xianyang, Shaanxi, China; ^2^College of Life Sciences, Northwest A&F University, Xianyang, Shaanxi, China

**Keywords:** *Caragana* *korshinskii*, CkREV, vessel development, drought tolerance, auxin

## Abstract

Drought stress poses severe threat to the development and even the survival status of plants. Plants utilize various methods responding to drought, among which the forming of more well-developed xylem in leaf vein in woody plants deserves our attention. Herein, we report a transcription factor CkREV from HD-ZIP III family in *Caragana korshinskii*, which possesses significant functions in drought response by regulating xylem vessel development in leaf vein. Research reveal that in *C. korshinskii* the expression level of *CkREV* located in xylem vessel and adjacent cells will increase as the level of drought intensifies, and can directly induce the expression of *CkLAX3*, *CkVND6*, *CkVND7*, and *CkPAL4* by binding to their promoter regions. In *Arabidopsis thaliana*, CkREV senses changes in drought stress signals and bidirectionally regulates the expression of related genes to control auxin polar transport, vessel differentiation, and synthesis of cell wall deposits, thereby significantly enhancing plant drought tolerance. In conclusion, our findings offer a novel understanding of the regulation of CkREV, a determinant of leaf adaxial side, on the secondary development of xylem vessels in leaf vein to enhance stress tolerance in woody plants.

## Introduction

Drought serves as one of abiotic stresses that commonly appear in northwest regions of China. It triggers water deficiency that poses severe threat to the survival and yield of plants (Wu et al., 2014). Shrub *Caragana korshinskii* from Fabaceae *Caragana* performs excellently against drought as it forms various distinct shapes and structures that endow itself with strong capability to adapt to the circumstances during natural evolution (Ma et al., 2016; Waseem et al., 2021). Plants utilize quantities of methods responding to drought, among which the forming of more well-developed conducting tissue so as to improve water use efficiency in woody plants deserves our attention (Sack and Scoffoni, 2013; Fletcher et al., 2018; Yao et al., 2021). Furthermore, acting as one of the organs that directly sense environmental changes and provide photosynthetic product, leaves are most sensitive to environmental changes so their drought adaptability is also under the spotlight.

Plant vascular system mainly consists of xylem, phloem, and cambium (Yamaguchi et al., 2010). Well-developed xylem is able to offer stronger mechanical support and ensure fluent water movement in order to further promote the capacity of water transport (Rogers and Campbell, 2004). Hence, plants can resist drought stress by the formation of more well-developed xylem, but how do plants achieve that under drought response? Being the developmental determinant of leaf adaxial side, HD-ZIP III family plays a broad regulatory role from the birth to the death of plants, which participates in embryogenesis, meristem maintenance, leaf and vascular bundle development, inflorescence structure, ovule development, growth response to environmental signals, and senescence (Prigge et al., 2005; Xie et al., 2014). We particularly concentrate on the significant function of HD-ZIP III family in leaf vascular development. In *Arabidopsis thaliana*, the number of stele vessels slightly decreased in mutant *rev-9* that lost the function of *REVOLUTA* (*REV*), a member from HD-ZIP III family, while the scenario was completely reversed in a gain-of-function mutant *rev-10d* (Ilegems et al., 2010; Hong et al., 2020). Although the ectopic expression of other four members in HD-ZIP III family, namely *PHABULOSA* (*PHB*), *PHAVOLUTA* (*PHV*), *CORONA/HOMEOBOX GENE15* (*CNA/ATHB15*), and *HOMEOBOX GENE 8* (*ATHB8*), exhibits corresponding phenotype changes, respectively; however, only individuals with a single mutation of *REV* display the phenotype distinct from wild-type *A. thaliana*, including defects in leaf and vascular bundle development as well as deficiency in auxin transport (Prigge et al., 2005; Ramachandran et al., 2017). Moreover, *in situ* hybridization of *OsHB1* homologous to *REV* in different developmental stages of rice leaves reveals that *OsHB1* possesses a broader localization compared with other family members (Itoh et al., 2008). The results above unveil that *REV* probably plays a regulatory role more critical than others.

Plant leaves develop in proximodistal, dorsoventral (adaxial-abaxial), and mediolateral patterns following initiation. The Myb domain gene ASYMMETRIC LEAVES1 (AS1) is required for adaxial fate in many plants (Waites et al., 1998; Tattersall et al., 2005). *AS1* overexpression producing a leaf curling phenotype was subsequently shown as a positive regulator of the expression of *PHB*, *PHV*, and *REV* (Xu et al., 2003; Fu et al., 2007). Such observations were consistent with phenotypes of dominant gain-of-function *phb-1d* and *phv-1d*, which had earlier been described as having amphivasal vascular bundles with xylem surrounding phloem (McConnell and Barton, 1998; McConnell et al., 2001). Genes of the KANADI family of GRAS-type transcription factors have been shown to play a role in the regulation of leaf polarity by promoting phloem development, which is opposite to that of HD-ZIP III family transcription factors (Ilegems et al., 2010). AS1, as a key transcription factor in the development of the adaxial pattern of leaves, inhibits the expression of KAN family genes by interacting with AS2, and promotes the development of the adaxial pattern of the leaves (Xu et al., 2003). Although many studies have described the close relationship between AS1 and the HD-ZIP III family, their interactions and regulatory mechanisms have not been reported.

Auxin is the earliest discovered plant hormone, which participates in numerous processes throughout plant growth and development (Zhao, 2010; Olatunji et al., 2017; Brumos et al., 2018; Bu et al., 2020). It goes hand in hand with xylem development (Xu et al., 2019; Wakatake et al., 2020), since its appropriate concentration is a prerequisite for the differentiation of vessel cells (Fukuda and Komamine, 1980). Meanwhile, auxin works on the basis of the polar transport co-mediated by its inflow and outflow transporter (Fàbregas et al., 2015). The formation of plant vascular system originates from precursor cells in procambium, whose differentiation to vessel cells in xylem requires the polar transport of auxin (Ohashi-Ito and Fukuda, 2010). In *A. thaliana*, REV positively regulates the expression of auxin inflow transporter gene *LAX2* and *LAX3* to guide its polar transport in favor of xylem development (Baima et al., 2014); nevertheless, its regulatory pattern under drought response has not been reported yet.

Apart from auxin, xylem development is also closely related to the biosynthesis of cellulose, lignin and xylan. Secondary cell wall accumulating on the inner side of the primary wall after cell growth stops serves as an essential condition for xylem vessel cells, tracheid, and sclerenchyma cell, etc. Cellulose is the frame of cell wall and several layers of newly-synthesized cellulose, hemicellulose, and lignin are deposited on the primary cell wall to build well-developed secondary wall (Meents et al., 2018). VNDs and NSTs belong to a subgroup of transcription factors with NAM/ATAF/CUC (NAC) domain, among which *VND6* and *VND7* are particularly considered as the chief regulator initiating vessel differentiation in metaxylem and protoxylem, respectively. A cascade regulation network on transcription is formed downstream of those regulators, controlling the expression of genes, such as cellulose synthase, responsible for secondary cell wall synthesis (Yamaguchi et al., 2010, 2011). Previous studies have shown that REV can induce the expression of *VND7* (Endo et al., 2015), while sometimes the situation may be different when VND7 conversely acts upstream of *REV* to promote or inhibit the expression of *REV*. This complex regulatory pattern may be closely related to external signals (Taylor-Teeples et al., 2015). The function of *VND6* and *VND7* in regulating vessel differentiation and secondary cell wall formation has been thoroughly decoded (Yamaguchi et al., 2010, 2011; Endo et al., 2015; Taylor-Teeples et al., 2015); nevertheless, their response to drought stress has not been reported yet.

Herein, we discovered that the expression of transcription factor CkREV from HD-ZIP III family was up-regulated and localized in xylem in *C. korshinskii* under drought stress. It directly regulated the expression of *CkLAX3*, *CkVND6*, *CkVND7*, and *CkPAL4* concerning the development of xylem vessels in leaf vein in a drought-level-dependent manner, which facilitated more well-developed xylem in leaf vein and significantly improved drought tolerance. To conclude, our findings offer novel insight into stress resistance pathways and the regulation mechanism thereof in woody plants.

## Materials and methods

### Plant materials

*Caragana korshinskii* employed in our research was selected from the natural rainfall gradients of Yangling (600 mm), Huangling (560 mm), Ansai (520 mm), Yulin (480 mm), Shenmu (440 mm), and the nursery garden in Northwest Agriculture and Forestry University (NWAFU) after approximately 10 years of growth under nature conditions. Healthy seeds were washed by clean water and settled in dark environment for germination at room temperature for 3–5 days wrapped by wet gauze until being transplanted into soil. Additionally, all the *A. thaliana* employed in our research belong to Col-0 ecotype, and detailed information is listed in the table of key resources. *A. thaliana* seeds were soaked in distilled water for vernalization at 4°C for 48–72 h prior to disinfection using 10% (*v*/*v*) NaClO. Disinfected seeds were washed by sterile water for 6–8 times before being placed separately on 1/2 MS solid culture medium (pH 5.7) with or without hygromycin at 50 μg/ml and residual water on the surface of seeds was dried up by filtered air in the clean bench. After the seedlings were cultured for 5–7 days, they were transplanted into soil or used for PEG-simulated drought treatment. A total of four gradients of 0, 2, 6, and 12 h were set. According to the different processing time, the *Arabidopsis* seedlings were transferred to the PEG medium in batches, and finally, the samples were collected uniformly.

As for transient transformation of *C. korshinskii* leaves, seedlings at the age of 30–40 days were employed as transformation material. *Agrobacterium tumefaciens* holding *p35S::CkREV-GFP* was cultured in LB medium with 50 mg/l kanamycin and 20 mg/l rifampicin at 28°C for a night in a shaking incubator and 0.5 ml of the culture was added into another 50 ml LB liquid medium the next day for shaking incubating at 28°C until its OD_600_ reached 0.8. After centrifugation at 4,000 rpm for 10 min, bacterium deposition was resuspended in 1/2 MS transformation solution containing 150 μM AS, 2.5% sucrose, and 0.01% (*v*/*v*) Tween 20 at pH 5.8 after OD_600_ was adjusted to 0.8. *C. korshinskii* seedlings were soaked in 1/2 MS hyperosmotic solution (25% sucrose, pH 5.8) for 2 h for hyperosmotic pretreatment, followed by treatment in transformation solution for 6 h. Then, seedlings were washed by distilled water twice and cultured for 66 h under normal light conditions until qRT-PCR analysis (Ji et al., 2014). As for drought treatment of *C. korshinskii*, after the last adequate watering, the watering was stopped for 7 days as LD, 14 days as MD, and 21 days as SD.

### qRT-PCR analysis

Primers for each gene in qRT-PCR were designed by Premier Primer 5.0 (Supplementary Tables 1, 2). Plant RNA Issolation Kit (Cat# 082002, BeiBei Bio) was utilized for the total RNA extraction and HiScript II Q RT SuperMix for qPCR (Cat#R223, Vazyme) was employed for the reverse transcription of cDNA. 10 μl qPCR system was combined by 5 μl mix, 0.25 μl F and R primers, 3.5 μl water, and 1 μl cDNA using 2 × M5 HiPer Real-time PCR Supermix (Cat#MF013, Mei5bio). Each treatment contains at least 3 biological replicates, and the relative expression of the gene was calculated by the 2 ^−ΔΔCT^ method with the expression of the *actin 2* gene as a reference (Li et al., 2022).

### RNA *in situ* hybridization

DIG RNA labeling kit (Cat#11277073910, Roche), T7 RNA polymerase (Cat#10881767001, Roche), and T3 RNA polymerase (Cat# 11031163001, Roche) were employed for *in vitro* transcription to obtain sense probe and antisense probe labeled by DIG after purification. Paraffin sections of *C. korshinskii* leaves were dewaxed and rehydrated through graded ethanol to pure water, according to a previous study (Kouchi and Hata, 1993), a series of pretreatments were performed on the sections, the probes were incubated with the sections, and the probe localization on the sections was stained and observed by NBT/BCIP.

### Immunohistochemical staining

Analysis of the three-dimensional structure of the CkREV protein shows the appropriate antigenic determinant contains about 8–12 amino acids, and after artificial synthesis, it was used as an antigen to immunize healthy New Zealand white rabbits by subcutaneous injection. After the rabbits were immunized for 4 times, blood was collected from heart and placed under constant temperature without disturbance until serum was collected as polyclonal antibody against CkREV protein. Immunohistochemical staining using SP method was performed after slides were naturally cooled down to room temperature. Eventually, DAB was added as chromogenic substance prior to photography in bright field (Chen et al., 2010). SP Rabbit HRP Kit (Cat#CW2069, CWBIO) provided several reagents in this experiment and the expression pattern of CkREV at tissue level was observed in our paraffin sections with the help of the primary antibody against it.

### Safranin staining

Baked sections were soaked into dimethylbenzene to dewax twice, each of which lasted 30 min. Then they were rehydrated by 100 and 95% ethanol for 5 min each. Safranin staining lasted 3–10 min. After washing with 95% ethanol, the tissue sections were observed under a microscope (Cabello and Chan, 2019).

### Toluidine blue staining

Toluidine blue (Cat# G3668, Solarbio) was diluted to working concentration 0.1% by distilled water. 40–100 μl solution was added to slides that went through dewaxing and graded rehydration (100, 95, 85, 70, and 50% ethanol and water; each concentration for 1 min) for 1–3 min. Then distilled water was used to wash the slides twice or three times. Ultimately, a piece of cover glass was put on the sample after a drop of distilled water was added. In bright field, toluidine blue staining contributed to purple primary cell wall and blue secondary cell wall (Cabello and Chan, 2019).

### Phloroglucinol staining

Lignin synthetic pathway mainly contributes to the production of H, G, and S monomer of lignin, among which G and H monomer can be stained by phloroglucinol. Tissue sections were set on slides with several drops of 1% phloroglucinol dissolved in 95% ethanol. Then, several drops of 12% hydrochloric acid were added a moment later. Ultimately, slides were sealed and observed under a microscope for photography (Liu et al., 2014).

### Lignin auto-fluorescence

Tissue sections were sealed by 50% glycerol and settled under a fluorescence microscope. Ultraviolet, blue, and green light were employed as excitation light source and fluorescence signals were recorded by photographs. Currently, it has been prevailing to detect the blue fluorescence excited by ultraviolet or the green fluorescence excited by blue light (Donaldson, 2020).

### Zinc iodide chloride method

Plant cellulose staining solution (Cat#R30129, Yuanye Bio) was employed for cellulose staining. Sections were dewaxed and rehydrated following the process mentioned in Toluidine Blue Staining. Staining solution was added on slides for 5 min, after which cellulose became purple blue or blue while lignin, cutin, and suberin appeared orange.

### Calcofluor white staining

Calcofluor white (Cat#18909, Sigma) mother liquid was diluted 50 times before staining. Appropriate amount of staining solution and 10% KOH were added on the slides for 3–5 min followed by washing with distilled water. Then, 50% glycerol was employed for tabletting before observation under ultraviolet. Calcofluor white binding to cellulose shone blue fluorescence (Yu and Zhao, 2012).

### Genome walking

Currently, no genome date of *C. korshinskii* has been published. As a result, all the genes were obtained through genome walking. To begin with, three specific primers were designed according to known sequence region, among which SP2 was designed within SP1 and SP3 was within SP2. It was advisable that primers should be close to 5′ end as much as possible and they were required not to be near to each other. Universal F primers were LAD1–LAD4 and AC1 (Supplementary Table 3). The steps and reaction procedures were carried out as described in the article (Liu and Chen, 2007).

### Dab staining

Diaminobenzine (DAB) acts as a coloring agent to detect the existence and distribution of hydrogen peroxide in plant cells since DAB can generate dark-brown precipitates oxidized by it. 1 mg/ml DAB solution was prepared and adjusted to pH 3.0 by 0.2 M HCl. 5 μl Tween 20 and 0.5 ml 200 mM Na_2_HPO_4_ were added into the DAB solution while stirring to get 10 mM Na_2_HPO_4_ DAB staining solution, which rose pH again. Collected leaves were soaked into DAB solution prior to vacuumization followed by shaking incubation for 4–5 h at 80–100 rpm under dark conditions. Ultimately, DAB solution was replaced by bleaching solution (ethanol:acetic acid:glycerol = 3:1:1) before photography (Daudi and O’Brien, 2012).

### Yeast two-hybrid

Cotransformed yeasts for self-activation verification were applied on solid SD/−Leu-Trp medium and cultured upside down at 30°C for 3–4 days. Healthy yeast colonies were selected and added to liquid SD/−Leu-Trp medium for shaking culture until OD_600_ value reached 1.0, followed by graded dilution into three concentrations. Later, diluted culture was applied on new solid SD/−Leu-Trp medium and SD/−Leu-Trp-His-Ade 3-AT medium and cultured upside down at 30°C for 3–4 days. Subsequent experiments would be carried out under 3-AT concentration that inhibited self-activation.

### Bimolecular fluorescence complementation

The CDS of *CkAS1* gene without stop codon was constructed on pSPYCE(M) vector while that of *CkREV* gene with stop codon was constructed on pSPYNE(R)173 vector. Bacteria solutions were mingled in a ratio of 1:1 and injected into the lower surface of tobacco leaves at the age of 4 weeks under normal conditions, followed by 1 day of dark culture and 1–2 days of normal culture. Regions around the injection site were collected and placed under a microscope for YFP fluorescence detection (Chen et al., 2013).

### LUC complementation imaging assays

The CDS region of two proteins was constructed on cLUC and nLUC vectors, respectively, followed by *A. tumefaciens* GV3101 transformation. Bacteria solutions were mingled in a ratio of 1:1 and injected into the lower surface of tobacco leaves at the age of 4 weeks under normal conditions, followed by 1 day of dark culture and 1–2 days of normal culture. The whole leaves were cut and 100–200 μl substrate of FLuc (Cat#FR101, TransGen) was applied on the back for dark treatment for 5 min. Ultimately, leaves were placed in living plant molecular marker imaging system (Lumazone Pylon 2048B, Princeton, American) to detect LUC activity (Tian et al., 2021).

### Dual-luciferase reporter system

The promoter region was linked to the 5′ end of FLUC on pGreen II 0800-LUC vector and RLUC driven by 35S promoter served as internal reference. Meanwhile, *pCambia 1*,*302-CkREV*, *pCambia 1*,*302-CkAS1*, and *pCambia 1*,*302-GFP* empty vector were used as effectors. Mediated by *A. tumefaciens* GV3101 (pSoup-p19), reporter genes and effector plasmids were mingled at different proportions and combinations and coinjected into *Nicotiana benthamiana* leaves at the age of 28 days. Regions around the injection site were collected 48 h after injection and ground, followed by activity detection on firefly luciferase and ranilla luciferase through multi-wavelength microplate reader with full function (Infinite M200pro, Tecan) using TransDetect^®^ Double-Luciferase Reporter Assay Kit (Cat#FR201, TransGen) following the instructions. There should be no less than three biological repeats for each group while experimental and control groups in one repeat were required to symmetrically distribute along the two sides of the midrib of one leaf (Yan et al., 2021).

### Electrophoretic mobility shift assay

Electrophoretic mobility shift assay (EMSA) was employed to detect *in vitro* binding of CkREV to the cis-acting element of target genes downstream of it. Firstly, single-stranded DNA probe was synthesized and labeled with biotin at 3′ end using biotin label kit (Cat#GS008, Beyotime), followed by biotin-labeled double-stranded probe synthesis through annealing. Secondly, the prepared probe was incubated with protein purified *in vitro* under room temperature while unlabeled probe (cold probe) in graded concentration was added into control group for competitive binding against the labeled one. Thirdly, native PAGE was applied to separate samples prior to color developing on completely cross-linked nylon membrane according to instruments of EMSA chemical luminescence detection kit (Cat#GS009, Beyotime). Ultimately, results were observed under chemiluminescence imager (Yan et al., 2021).

### Yeast one-hybrid

Promoters of genes requiring interaction confirmation were constructed on pHIS2 vector for self-activation verification, respectively. Cotransformed yeasts for self-activation verification were applied on solid SD/−Leu-Trp medium and cultured upside down at 30°C for 3–4 days. Healthy yeast colonies were selected and added to liquid SD/−Leu-Trp medium for shaking culture until OD_600_ reached 1.0, followed by graded dilution into three concentration. Later, diluted culture was applied on new solid SD/−Leu-Trp medium and graded SD/−Leu-Trp-His/3-AT medium. Growth status of yeast cells was observed after 3–4 days of culturing at 30°C upside down. Subsequent experiments would be carried out under 3-AT concentration that inhibited self-activation (Hickman et al., 2013).

### Accession numbers

These sequence data have been submitted to the GenBank databases under accession number *CkREV*(MZ720786), *proCkLAX3*(MZ810547), *proCkVND6*(MZ810550), *proCkVND7*(MZ810551), *proCkPAL4*(MZ810549), and *proCkWRKY53*(MZ810548). Addresses are as follows: GenBank, http://www.ncbi.nlm.nih.gov.

## Results

### Drought stimulates more well-developed conducting tissue in *Caragana korshinskii* leaves

**Figure 1:**
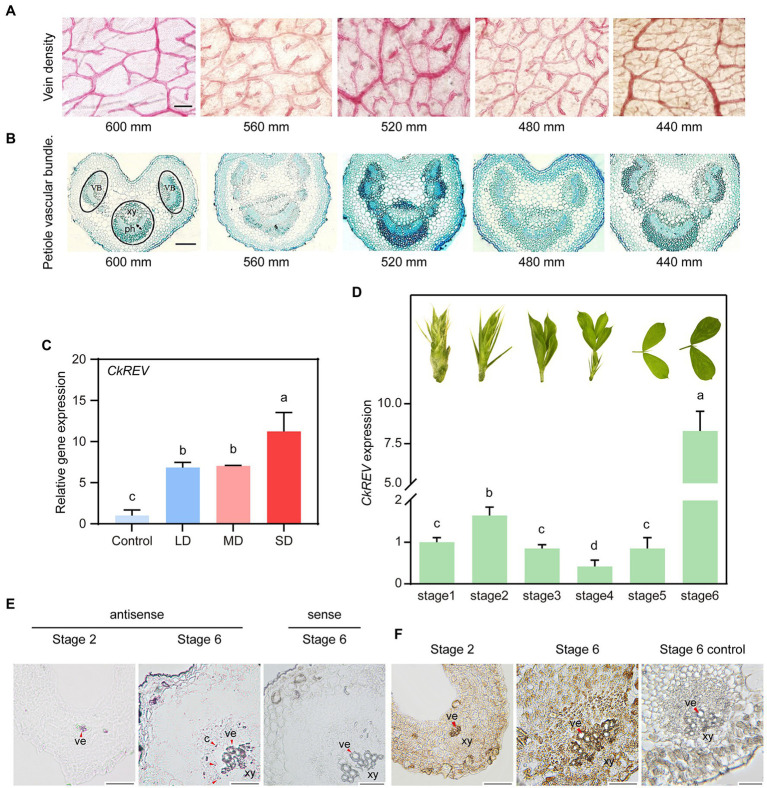
Drought induces the secondary development of conducting tissue in *Caragana korshinskii* leaves and the analysis on *CkREV* expression pattern. **(A)** Observation of vein density of *C. korshinskii* leaves under different rainfall gradients. **(B)** Observation of vascular bundle morphology in *C. korshinskii* petiole sections under different rainfall gradients. **(C)** qRT-PCR analysis on the expression level of *CkREV* after *C. korshinskii* was treated with natural drought for different durations. LD, light drought; MD, moderate drought, SD, Severe drought. **(D)** The development of *C. korshinskii* is divided into Stage1-Stage6 and qRT-PCR is performed on leaves of different stages to test *CkREV* level. Pictures above each column indicate leaf shape of that stage. **(E)** Tissue distribution of *CkREV* among different developmental stages of vein detected by RNA *in situ* hybridization. Purple signal signifies positive and red arrows are employed to indicate the position. ve, vessel; c, cambium; xy, xylem. **(F)** Tissue distribution of CkREV protein among different developmental stages of vein detected by immunohistochemical staining. Brown signal signifies positive and red arrows are employed to indicate the position. ve, vessel; xy, xylem; mm, stands for millimeters; which is the unit of rainfall. Error bars indicate SD (*n* = 3). **(C**,**D)** One-way ANOVA is performed for the statistical analysis, where different letters represent significant differences (*p* < 0.05). Scale bars in **(A**,**B**,**E**,**F)** = 50 μm.

We collected *C. korshinskii* leaves for safranin staining from diverse rainfall areas in the loess plateau in order to observe their changes in vein density. Results showed that vein density was inclined to increase as precipitation decreased (Figure 1A). Additionally, we performed toluidine blue staining on the sections of petiole from different rainfall areas to discover that the vascular bundle in leaf vein became more in amount and larger in volume with lignified cells, especially sclerenchyma cells and vessel cells, inclined to a well-developed stage as precipitation dropped, namely drought level intensified (Figure 1B).

### *CkREV* is induced by water deficiency and localized in xylem region

*REV* is among five HD-ZIP III genes, whose mutated phenotype first depicts the consequence of loss-of-function HD-ZIP III mutants (Talbert et al., 1995). Protein domain and phylogeny analysis revealed that CkREV shared the same conservative domain with AtREV and was distinct from other family members in a relatively independent clade (Supplementary Figures 1A,B). Present research regarding *REV* have largely focused on its regulation on normal development while little attention has been paid to its response under stress condition. We provided natural drought conditions for healthy *C. korshinskii* seedlings and utilized qRT-PCR to determine the expression level of *CkREV*. Results indicated that *CkREV* was induced under drought stress and the level thereof continued to rise as drought degree intensified (Figure 1C).

We further explored the expression pattern of *CkREV* among different development stages of leaves. The level of *CkREV* was measured after RNAs were extracted in different development stages (stage 1-stage 6) of *C. korshinskii* leaves (Figure 1D). We selected leaves at stage 2 and stage 6 bearing the highest level of *CkREV* for RNA *in situ* hybridization to demonstrate its tissue localization in leaf veins, the results of which exhibited that *CkREV* was expressed in xylem vessels in both stage 2 and stage 6 (Figure 1E and Supplementary Figure 2). Furthermore, *CkREV* also possessed a broader distribution in stage 6 compared with stage 2, whose positioning signals appeared in vessel precursor cells, xylem parenchyma cells, and finitely in cambium (Figure 1E). We made further efforts to detect the tissue localization of CkREV protein in stage 2 and stage 6 in vascular bundle in leaf vein by means of immunohistochemical staining so as to verify the authentic function of CkREV, whose results revealed that the positioning signal of CkREV was partially lost in cambium (Figure 1F).

### CkREV stimulates the formation of more well-developed conducting tissue in plants

**Figure 2:**
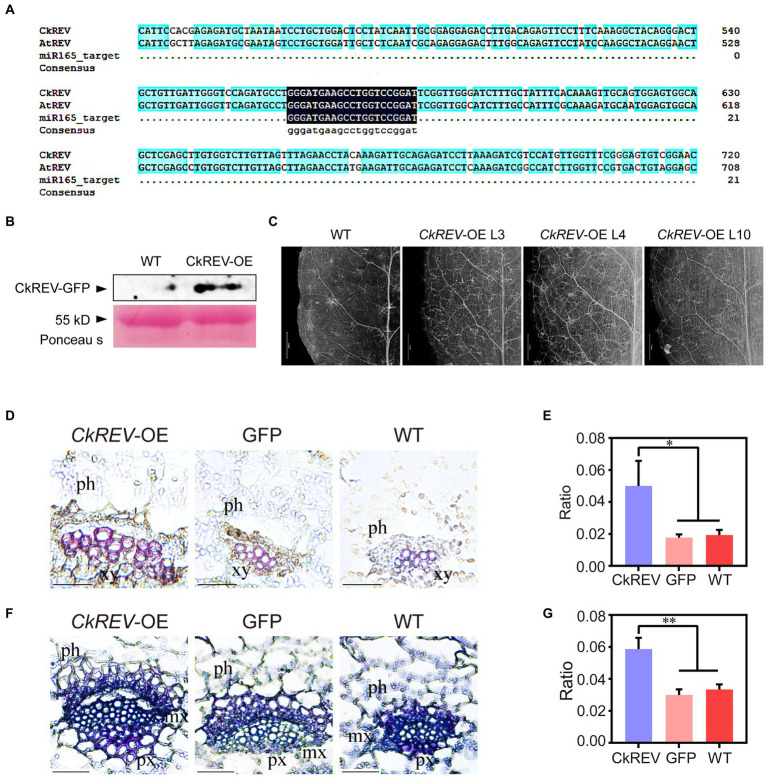
CkREV facilitates more well-developed conducting tissue in *Arabidopsis thaliana.* **(A)** Sequence alignment. **(B)** Detection of CkREV proteisn expression. **(C)** Observation of vein density of *A. thaliana CkREV*-OE lines. Scale bar = 1 mm. **(D,E)** Safranin staining of vein sections of *A. thaliana CkREV*-OE lines **(D)** and the ratio of stained xylem region to the entire vascular bundle **(E)**. ph, phloem; xy, xylem. **(F,G)** Toluidine blue staining of vein sections of *A. thaliana CkREV*-OE lines **(F)** and the ratio of stained xylem region to the entire vascular bundle **(G)**. ph, phloem, px, protoxylem, mx, metaxylem. Error bars indicate SD (*n* = 3). **(E,G)** Student’s *t*-test is employed to measure statistical significance between two samples with confidence level at 0.95 (^*^p < 0.05; ^**^p < 0.01). Scale bars in **(D,F)** = 50 μm.

CkREV was overexpressed in *A. thaliana* (*CkREV-*OE) for function verification. In order to clarify its real regulation as much as possible, we did not mutate the target sequence of miR165 (Figure 2A), and detected the protein expression level of CkREV in transgenic plants by western blot. The results showed that CkREV protein can be normal expressed (Figure 2B). It turned out that the vein density of *CkREV*-*OE* lines was inclined to rise compared with that of wild-type *A. thaliana* (Figure 2C). Paraffin section and safranin staining were also performed on transgenic *A. thaliana*, which unveiled that the xylem in vascular bundle tended to be well-developed with larger volume and significantly more vessels (Figures 2D,E). Interestingly, *A. thaliana CkREV*-OE lines showed an increase in the amount of vessels both in protoxylem and metaxylem in contrast to the GFP-overexpression (*GFP-*OE) lines and the wild type (Figures 2F,G). After consulting previous published research, we can conclude that CkREV not only participates in the cell differentiation of metaxylem vessels, but also relates to the development of protoxylem vessels, which stimulates more well-developed conducting tissue in *A. thaliana*.

### CkREV promotes cellulose and lignin deposition in the xylem of *Arabidopsis* leaf veins by regulating the expression of related genes

**Figure 3:**
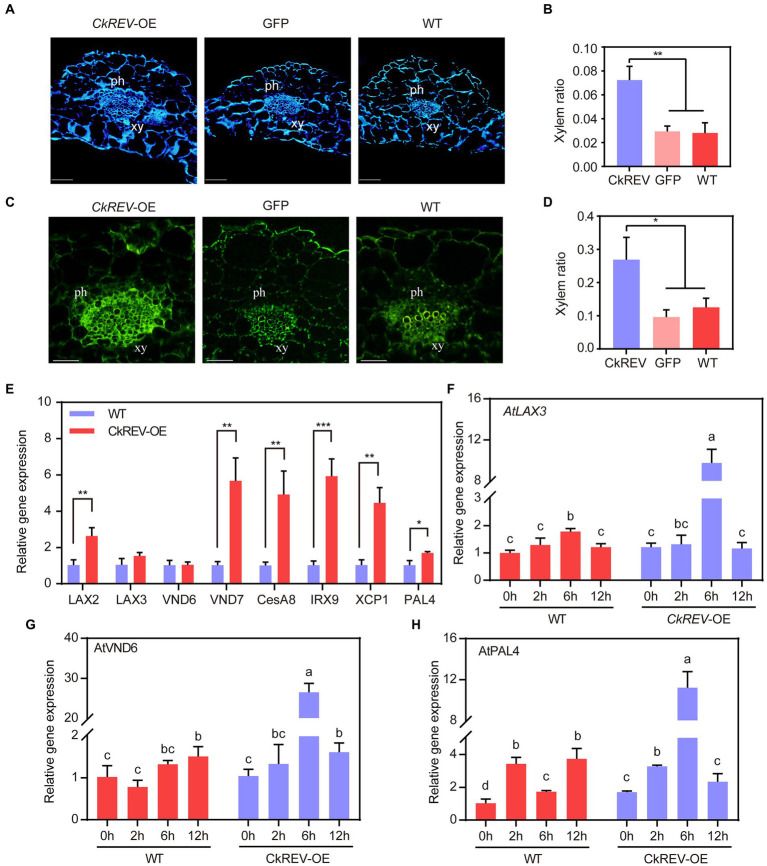
CkREV promotes cellulose and lignin deposition in xylem vessels. **(A,B)** Calcofluor white staining on leaf vein sections of *A*. *thaliana CkREV*-OE lines **(A)** and the ratio of stained xylem region to the entire vascular bundle **(B)**. ph, phloem; xy, xylem. **(C,D)** Observation on lignin auto-fluorescence on leaf vein sections of *A. thaliana CkREV*-OE lines **(C)** and the ratio of stained xylem region to the entire vascular bundle **(D)**. ph, phloem; xy, xylem. **(E)** qRT-PCR analysis on the level of genes concerning polar auxin transport, vessel differentiation, and the synthesis of cellulose, xylan, and lignin in *A. thaliana CkREV*-OE lines. **(F)** Expression pattern of *AtLAX3* gene detected by qRT-PCR under drought gradient. **(G)** Expression pattern of *AtVND6* gene detected by qRT-PCR under drought gradient. **(H)** Expression pattern of *AtPAL4* gene detected by qRT-PCR under drought gradient. Error bars indicate SD (*n* = 3). **(B,D,E)** Student’s *t*-test is employed to measure statistical significance between two samples with confidence level at 0.95 (^*^p < 0.05; ^**^p < 0.01; and ^***^p < 0.001). **(F–H)** One-way ANOVA is performed for the statistical analysis, where different letters represent significant differences (*p* < 0.05).

The deposition of cellulose and lignin is necessary for the formation of more developed xylem. To elucidate their deposition in transgenic *Arabidopsis*, cellulose in leaf vein sections was stained by the calcofluor white and zinc iodide chloride method, and lignin in leaf vein sections was stained by lignin autofluorescence and phloroglucinol. The deposition of cellulose and lignin was significantly increased in the vascular bundle xylem of leaf veins of *CkREV*-OE line compared to *GFP*-OE line and WT (Figures 3A–D and Supplementary Figure 3). The expression levels of related genes in *Arabidopsis CkREV*-OE line, *GFP*-OE line, and wild type were detected by qRT-PCR. *AtVND7*, *AtXCP1*, and *AtCesA8* associated with protoxylem vessel cell differentiation and cellulose synthesis, *AtIRX9* associated with xylan synthesis, and *AtPAL4* associated with lignin biosynthesis were significantly up-regulated, but the expression level of *AtVND6* associated with metaxylem vessel differentiation did not change obviously (Figure 3E). In addition to the above genes, polar transport of auxin also plays an important role in the secondary development of xylem. qRT-PCR found that the *AtLAX2* gene, which is related to the polar transport of auxin, was significantly up-regulated in the *CkREV*-OE line while the expression level of *AtLAX3*, similar to *AtVND6*, did not show significant changes (Figure 3E). The transgenic *Arabidopsis* was further subjected to drought treatment. Interestingly, *AtLAX3* and *AtVND6*, which were not induced by CkREV under normal culture conditions, and *AtPAL4*, which was slightly induced, were significantly up-regulated by CkREV with increasing degrees of drought compared to those of wild type under the same treatment (Figures 3F–H). Since the expression of *CkREV* is driven by the *CaMV 35S* promoter, this regulatory shift of CkREV under drought conditions is related to its signal sensing at the protein level rather than its changes at the transcriptional level.

### CkREV interacts with *CkLAX3*, *CkVND6*, *CkVND7*, and *CkPAL4* and induces their expression

**Figure 4:**
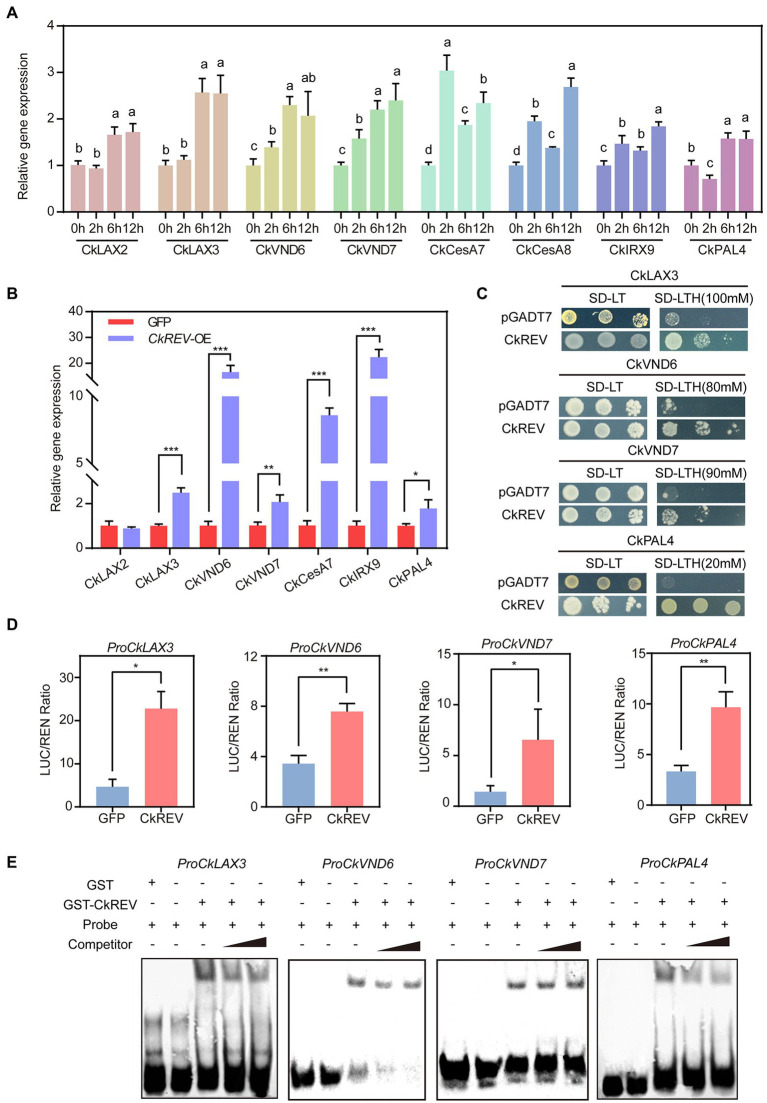
CkREV directly regulates the expression of *CkLAX3*, *CkVND6*, *CkVND7*, and *CkPAL4* to promote secondary development of vessels. **(A)** qRT-PCR analysis on the level of genes concerning polar auxin transport, vessel differentiation, and the synthesis of cellulose, xylan, and lignin after PEG-simulated drought treatment for corresponding durations on hydroponic *C. korshinskii* seedlings. **(B)** Expression levels of genes related to polar auxin transport, vessel differentiation, cellulose, xylan, and lignin synthesis in *CkREV* overexpressed *C. korshinskii* leaves. **(C)** The regulation of CkREV on *CkLAX3*, *CkVND6*, *CkVND7*, and *CkPAL4* tested by Y1H. **(D)** The regulation of CkREV on *CkLAX3*, *CkVND6*, *CkVND7*, and *CkPAL4* tested by Dual-LUC. **(E)** The regulation of CkREV on *CkLAX3*, *CkVND6*, *CkVND7*, and *CkPAL4* tested by EMSA. Error bars indicate SD (*n* = 3). **(A)** One-way ANOVA is performed for the statistical analysis, where different letters represent significant differences (*p* < 0.05). **(B,D)** Student’s *t*-test is employed to measure statistical significance between two samples with confidence level at 0.95 (^*^p < 0.05; ^**^p < 0.01; and ^***^p < 0.001).

Previous studies in *Arabidopsis* showed that under normal culture conditions, CkREV promotes xylem development by inducing the expression of *AtVND7*, *AtXCP1*, *AtCesA8*, *AtIRX9*, *AtPAL4*, and *AtLAX2*. Under drought conditions, CkREV can sense drought stress at the protein level, induce the expression of *AtVND6*, *AtPAL4*, and *AtLAX3*, and promote the secondary development of xylem. In order to better clarify the regulation mode of CkREV in *C. korshinskii*, drought stress was applied to *C. korshinskii*. With the intensification of drought gradient, the expression levels of the above-mentioned genes related to xylem development showed varying degrees of up-regulation (Figure 4A). Meanwhile, CkREV was overexpressed in *C. korshinskii* leaves by means of plasmolysis and restoration, and the overexpression of CkREV significantly induced the expression of the above genes except *CkLAX2* (Figure 4B). To further explore their targeting relationship, the promoter sequences of *CkLAX3*, *CkVND6*, *CkVND7*, and *CkPAL4* were cloned by genome walking. Yeast one-hybrid (Y1H), dual-luciferase reporter system (Dual-LUC), and EMSA show that CkREV interacts with *CkLAX3*, *CkVND6*, *CkVND7*, and *CkPAL4* and can directly bind to their promoter regions to induce their expression (Figures 4C–E and Supplementary Figure 4).

### CkREV negatively regulates the expression of downstream genes by interacting with CkAS1

**Figure 5:**
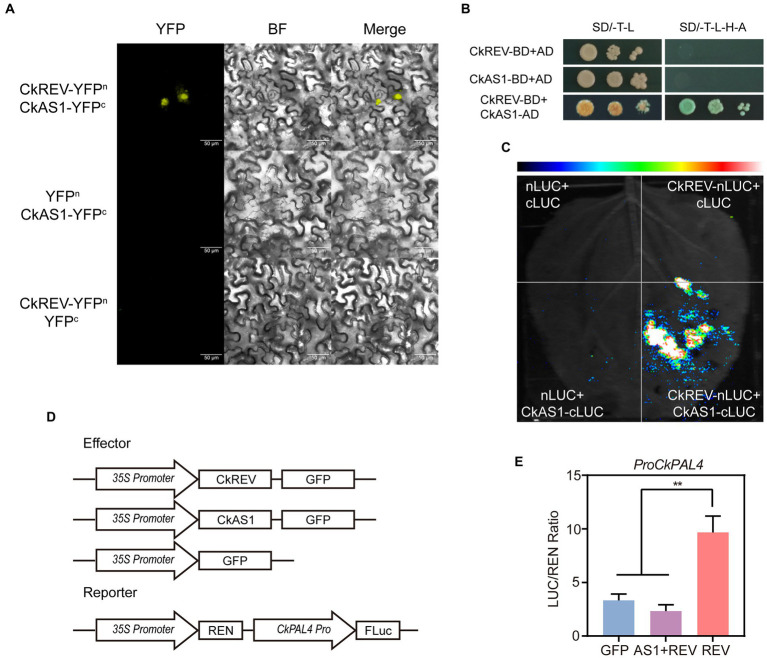
CkAS1 is involved in the negative regulation of downstream genes by CkREV. **(A)** BiFC assay for CkREV and CkAS1 interaction. **(B)** Y2H assay for CkREV and CkAS1 interaction. **(C)** LCI assay for CkREV and CkAS1 interaction. **(D)** Pattern graph of DUAL-LUC assay on the regulation of CkREV and CkAS1 on *CkPAL4.* **(E)** The interaction of CkREV with CkAS1 inhibits the induction of downstream genes by CkREV. Error bars indicate SD (*n* = 3). **(E)** Student’s *t*-test is employed to measure statistical significance between two samples with confidence level at 0.95 (^**^p < 0.01).

In *A. thaliana*, PAL family consists of four members, namely *PAL1*, *PAL2*, *PAL3*, and *PAL4*, among which *PAL1* and *PAL2* are apt to promote flavonoid synthesis while *PAL4* tends to boost lignin biosynthesis (Ro and Douglas, 2004; Olsen et al., 2008). We previously performed simulated drought on *A. thaliana CKREV*-OE lines using PEG at different concentrations so as to determine the expression level of *AtPAL4* through qRT-PCR and found that CkREV regulated *AtPAL4* in the four stages of drought in order of positive regulation, non-regulation, positive regulation, and negative regulation. The positive regulation of *AtPAL4* by CkREV disappeared after 2 h of mild drought (Figure 3H). This may be related to the response of flavonoids in the early stages of stress. The flavonoid pathway and the lignin pathway are two branches of the phenylpropane metabolic pathway and play important roles in the effective scavenging of ROS produced under drought stress. The results of qRT-PCR showed that the expression levels of genes related to flavonoid biosynthesis were significantly up-regulated in the *CkREV*-OE line (Supplementary Figure 5) and CkREV also affected the flavonoid metabolic pathway. Interestingly, at the later stages of drought stress, the regulation of *AtPAL4* by CkREV changed from positive to negative regulation (Figure 3H). The results of the dual-luciferase reporter system showed that CkREV mainly acts as a transcriptional activator. Therefore, the negative regulation of downstream genes by CkREV requires the assistance of the remaining transcription factors. As another key transcription factor in xylem development, CkAS1 mainly functions as a transcriptional repressor. The protein interactions between them were verified by bimolecular fluorescence complementation (BIFC), yeast two-hybrid (Y2H), and LUC complementation imaging (LCI; Figures 5A–C), and the interaction of CkAS1 with CkREV significantly inhibited the activation of *CkPAL4* by CkREV (Figures 5D,E). It is beneficial to reduce the deposition of lignin in the vessel and reduce the loss of water when water cannot be obtained from the external environment in the later stages of drought stress, thereby further enhancing the drought tolerance of the plant.

### *Arabidopsis thaliana CkREV*-*OE* lines is highly competent to eliminate ROS and accommodate to drought

**Figure 6:**
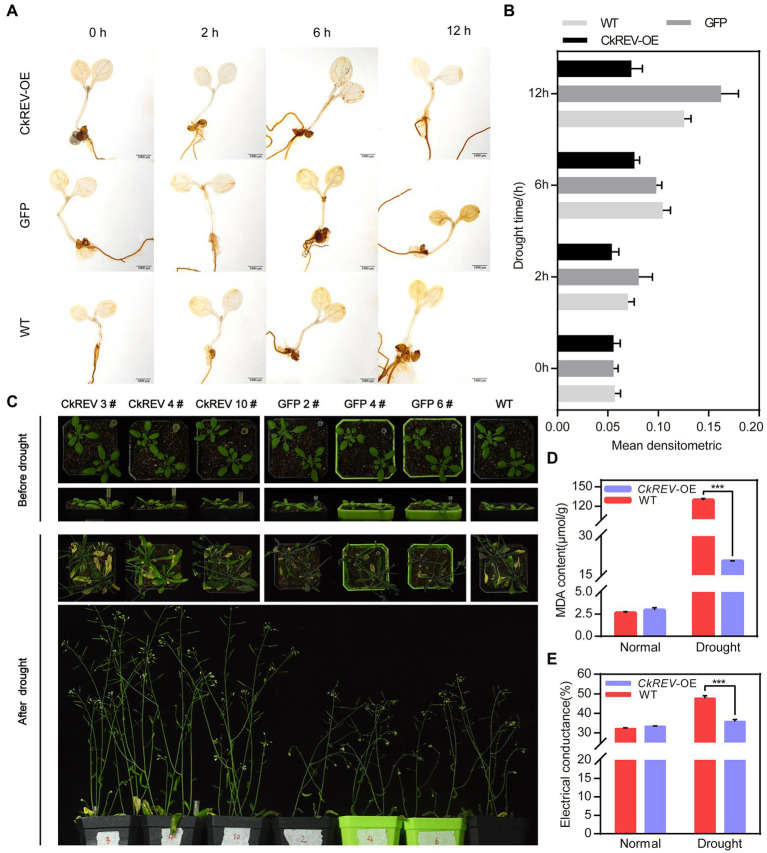
CkREV significantly enhances drought tolerance in plants by facilitating xylem development in leaf veins. **(A)** *Arabidopsis thaliana CkREV*-OE lines, *GFP-OE* lines, and the wild-type seedlings are placed on PEG medium for simulated drought treatment for corresponding durations prior to DAB staining on ROS within them and darker brown indicates higher ROS content. Scale bar = 1 mm. **(B)** Statistical analysis of mean densitometric analysis of ROS staining in *A. thaliana* after drought treatment. **(C)** Observation on living status of *A. thaliana CkREV*-OE lines, *GFP*-OE and the wild type after natural drought treatment. **(D,E)** Physiological indices determination on transgenic *A. thaliana*, malondialdehyde content **(D)**, and relative conductivity measurement **(E)**. Error bars indicate SD (*n* = 3). **(D,E)** Student’s *t*-test is employed to measure statistical significance between two samples with confidence level at 0.95 (^***^p < 0.001).

CkREV facilitates more advanced xylem vessels by numerous pathways under drought response. For the sake of a better interpretation of the improvement in drought tolerance derived from well-developed leaf veins in plants, drought treatment was imposed on *A. thaliana CkREV*-OE, *GFP*-OE, and wild-type lines. It was observed after DAB staining that *CkREV*-OE lines retained less reactive oxygen species (ROS) compared to *GFP*-OE lines and the wild type under increasing drought degree (Figures 6A,B). Afterward, natural drought was performed on lines mentioned above, in which *CkREV*-OE lines were taller with more lateral branches and relatively good leaf growth status compared to others (Figure 6C). Meanwhile, we measured physiological indices of drought-treated *A. thaliana* to discover that *CkREV*-OE lines kept low content of malondialdehyde (MDA) with minor damage on cell membrane, which indicated that it suffered milder stress, exhibiting exceptional competence accommodating to drought (Figures 6D,E).

## Discussion

**Figure 7:**
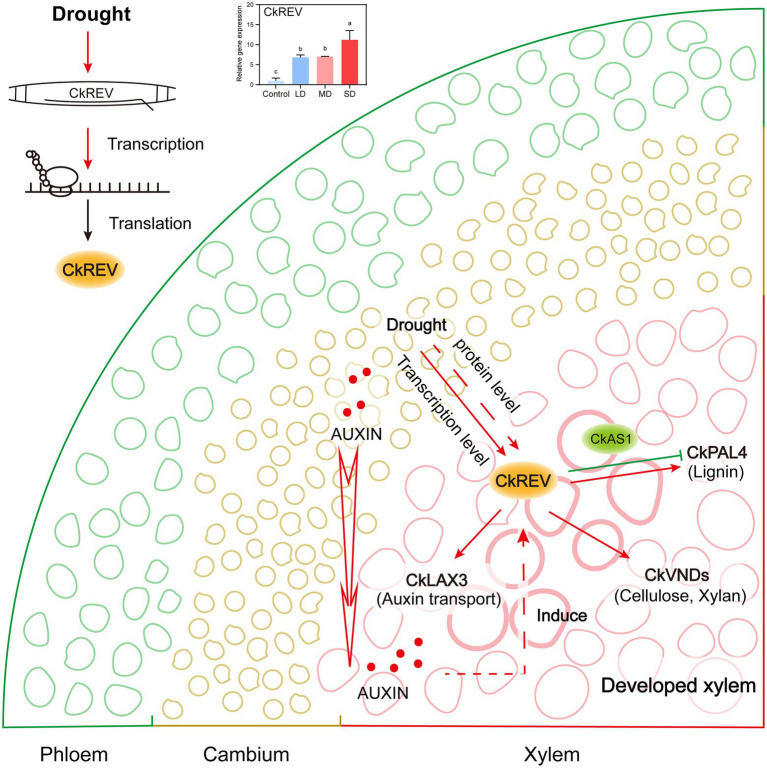
Model of CkREV regulating the secondary development of xylem vessels in response to drought. This model summarizes our results and shows that the expression level of CkREV located in xylem vessel and adjacent cells thereof will rise as the level of drought intensifies. CkREV senses drought stress signals, and regulates the expression of *CkLAX3*, *CkVND6*, *CkVND7*, and *CkPAL4* to control auxin polar transport, vessel differentiation, and synthesis of cell wall deposits, which is conducive to better plant growth under the condition of maintaining high drought tolerance.

Woody plants living in the northwest regions in China serve as preferable samples for studies focusing on drought adaptability. The formation of more well-developed leaf veins against drought stress plays an indispensable role in responding to water deficiency for woody plants, which deserves our attention in particular (Sack and Scoffoni, 2013; Fletcher et al., 2018; Yao et al., 2021); nevertheless, current interpretations are insufficient on its mechanism. Herein, we report a dominant species *C. korshinskii* used for vegetation restoration in The Loess Plateau, whose transcription factor CkREV from HD-ZIP III family not only works in plant normal growth but also actively participates in the regulation on secondary development of xylem in veins in response to drought (Figure 7).

### *CkREV* is induced by drought stress and promotes vessel development in xylem

*CkREV* can be induced by drought stress and possesses space fundamental to control xylem development under drought stress. RNA *in situ* hybridization and immunohistochemical staining revealed that CkREV shared analogous expression pattern with the homologous genes thereof in *Oryza sativa* and *Zinnia elegans*, which were all specifically localized in vessel cells, vessel precursor cells, and parenchyma cells in the xylem of leaf vein (Figures 1E,F; Ohashi-Ito and Fukuda, 2003; Itoh et al., 2008), implying it was probably involved in the regulation on secondary development of xylem in *C. korshinskii* veins in response to drought.

CkREV endows plants with more robust drought adaptability by means of facilitating more advanced conducting tissue. It is clear in preceding reports that HD-ZIP III family controls the differentiation of protoxylem and metaxylem in an expression-level-dependent manner since high level of its family members remarkably promotes the development of metaxylem in *A. thaliana* (McConnell and Barton, 1998; McConnell et al., 2001; Mallory et al., 2004; Zhong and Ye, 2004; Carlsbecker et al., 2010; Ilegems et al., 2010; Ramachandran et al., 2021). Interestingly, unlike *AtREV*, *CkREV* is competent in promoting the differentiation of vessels in both protoxylem and metaxylem in *A. thaliana* veins. More well-developed conducting tissue prominently elevates the drought adaptability of transgenic *A. thaliana* holding CkREV, including a healthier living state and greater stress resistance, which indicates that CkREV is of paramount importance under drought stress (Figure 6).

### CkREV offers auxin environment for xylem development in veins

Recent years have witnessed that not only abscisic acid, salicylic acid, and ethylene but also auxin engages in drought response (Rowe et al., 2016). Auxin is in close relationship with the secondary development of xylem (Ursache et al., 2014), whose function is based on the polar transport collectively supported by its inflow and outflow carriers (Fàbregas et al., 2015). In *A. thaliana*, AUX/LAX family consists of *AUX1* and *LIKE-AUX1* genes, including *LAX1*, *LAX2*, and *LAX3*, while evidence manifests that auxin carriers of AUX/LAX family are localized in vascular bundles and serve as inflow carriers (Yang et al., 2006; Péret et al., 2012; Baima et al., 2014). It is also clear that *AUX1* is of paramount significance in root gravitropism, lateral root and root hair development, phyllotaxy, and apical hook expansion (Swarup et al., 2008; Monshausen et al., 2011), that *LAX1* is necessary for phyllotaxy pattern (Bainbridge et al., 2008), that *LAX2* is responsible for the vascular configuration of cotyledon and vein pattern (Péret et al., 2012; Moreno-Piovano et al., 2017), and that *LAX3* is closely related to lateral root formation and apical hook development (Swarup et al., 2008). Preceding research have demonstrated that the expression level of members from AUX/LAX family in *A. thaliana* stems rises remarkably with auxin significantly concentrated in vascular bundles, which facilitates more advanced stem vascular bundles under gravity stress (Cabello and Chan, 2019). We noticed that *CkLAX2* and *CkLAX3* genes that encode auxin influx carriers were both up-regulated in *C. korshinskii* leaves as drought intensified. After CkREV was overexpressed in *C. korshinskii*, the expression level of *CkLAX3* was up-regulated while the expression level of *CkLAX2* did not change significantly. However, in the *Arabidopsis CkREV*-OE line under normal culture conditions, the expression of *AtLAX2* was up-regulated while the expression of *AtLAX3* did not change significantly. After drought treatment, *AtLAX3*, which was not induced by CkREV, was significantly up-regulated. The possible reason is that the transient transformation of *C. korshinskii* is accompanied by osmotic stress, which is similar to drought stress. The above results suggest that maybe *LAX2* is involved in the development of protoxylem, while *LAX3* is involved in the development of metaxylem. Promoter *CaMV 35S* initiated the expression of CkREV in our *A. thaliana CkREV*-OE lines; nevertheless, CkREV up-regulated *AtLAX3* as drought degree grows, which indicated it was the protein level rather than the transcription level of CkREV that dominated the regulation. Since the detailed mechanism was nebulous, we speculated that such regulation probably relied on the START domain on CkREV as it could bind to small hydrophobic molecules such as steroids, phospholipid, and carotenoids (Ponting and Aravind, 1999; Schrick et al., 2004). To conclude, CkREV influences the polar transport of auxin by regulating the expression of *CkLAX3* under drought conditions, which further controls vessel development in xylem.

### CkREV directly regulates vessel development while facilitating secondary metabolite deposition on vessel wall

*VND6* and *VND7* are recognized as the chief regulator initiating vessel differentiation in metaxylem and protoxylem, respectively. There is a cascade regulatory network on transcription downstream of them controlling genes regarding the construction of secondary wall, such as cellulose synthase, etc. (Yamaguchi et al., 2010, 2011). In *C. korshinskii*, the level of *CkVND6*, *CkVND7*, and *CkPAL4* was up-regulated as drought stress was growingly severe while *CkREV* overexpression significantly induced *CkVND6* and *CkPAL4* (Supplementary Figures 4, 6). Previous studies have demonstrated that AtREV can bind to the promoter region of *AtVND7* and enhance the expression of the latter (Endo et al., 2015) while sometimes the scenario can be different that *AtVND7* functioning upstream of *AtREV* inhibits *AtREV* by binding to its promoter region as well, which stringently controls the biosynthesis of lignin, etc. (Taylor-Teeples et al., 2015). We found that the *Arabidopsis CkREV*-OE line had more developed xylem vessel cells with significantly increased deposition of cellulose and lignin (Figures 3A–D and Supplementary Figure 3). When analyzing the expression levels of related genes in transgenic *Arabidopsis*, it was found that the expression levels of *AtVND7*, *AtXCP1*, *AtCesA8*, *AtIRX9*, and *AtPAL4* which were related to vascular development were all up-regulated. Similar to *AtLAX3*, the expression levels of *AtVND6* not induced by CkREV and *AtPAL4* slightly induced under normal culture conditions were up-regulated by CkREV more than 20-fold and 10-fold, respectively, with the aggravation of drought (Figure 3), possibly because ROS signals generated by drought stress were sensed by CkREV. In previous reports, the perception of ROS by the PAS domain on REV protein is required for *WRKY53* to be fully activated in the presence of ROS (Xie et al., 2014). In conclusion, our study further confirms that the regulation of downstream genes by CkREV is closely related to drought.

In the later period of enduring drought, moisture content was tremendously scarce for plants to absorb. Accordingly, it was salient for plants to cut down water loss so as to improve drought resistance. The tracheid cell wall and polysaccharide compounds in xylem vessel cells are illustrious for their strong hydrophilicity in vascular plants. Lignin cross-links polysaccharide in cell wall to attenuate its water absorption in favor of efficient water transport (Huang et al., 2010). Additionally, we found that CkREV switched to down-regulate the expression of *AtPAL4* at the later stages of drought, which was beneficial to reduce the biosynthesis of lignin in the xylem vessels and the polymerization level of polysaccharide in vessels and tracheids, thus weakening water transport, preventing water from rapidly draining away from vessels and further enhancing drought tolerance of plants. In *C. korshinskii*, the negative regulation of downstream genes by CkREV may be related to the mechanism of endogenous RNA degradation mediated by CkAS1, because the interaction of CkAS1 with CkREV significantly inhibited the activation of CkPAL4 by CkREV (Figures 5D,E).

In conclusion, from xylem development and stoma movement stages two aspects, this paper demonstrates that *C. korshinskii*, the dominant shrub in desert area, forms more advanced xylem. It is an innovative discovery that woody plants are endowed with drought tolerance mechanisms targeting diverse organs by a single gene. That is to say, CkREV localized in xylem region directly regulates *CkLAX3*, *CkVND6*, *CkVND7*, and *CkPAL4* responsible for vessel development in vein xylem in a manner depending on drought degree when *C. korshinskii* is confronted with drought stress, which contributes to well-developed xylem in vein. In conclusion, our research concentrating on REV offers novel insights into mechanisms of stress resistance in plants, more importantly, we seek out gene reference for resistant variety breeding for biomass and yield improvement under stress conditions in woody plants, such as tea plants.

## Data availability statement

The datasets presented in this study can be found in online repositories. The names of the repository/repositories and accession number(s) can be found in the article/Supplementary material.

## Author contributions

CG and JL designed the experiments in this study. JL accomplished the majority of experiments and writing of this manuscript. LX finished the identification and screening of transgenic plants and partial vector construction. JR and ZB completed the culture of experiment materials and promoter cloning of all the genes through genome walking. TZ, WZ, and JB were involved in the discussion of experimental ideas and performed data analysis. JC was responsible for the translation of the entire manuscript. All authors contributed to the article and approved the submitted version.

## Funding

This research was funded by the National Natural Science Foundation of China (nos. 31770648 and 31070538).

## Conflict of interest

The authors declare that the research was conducted in the absence of any commercial or financial relationships that could be construed as a potential conflict of interest.

## Publisher’s note

All claims expressed in this article are solely those of the authors and do not necessarily represent those of their affiliated organizations, or those of the publisher, the editors and the reviewers. Any product that may be evaluated in this article, or claim that may be made by its manufacturer, is not guaranteed or endorsed by the publisher.
