# Supplementary material for: CkREV regulates xylem vessel development in Caragana korshinskii in response to drought
Source: Front Plant Sci. 2022 Aug 25;13:982853. doi: 10.3389/fpls.2022.982853 (PMC9453446; doi:10.3389/fpls.2022.982853)
Supplement: Supplementary file 1 [file Data_Sheet_1.docx]

**
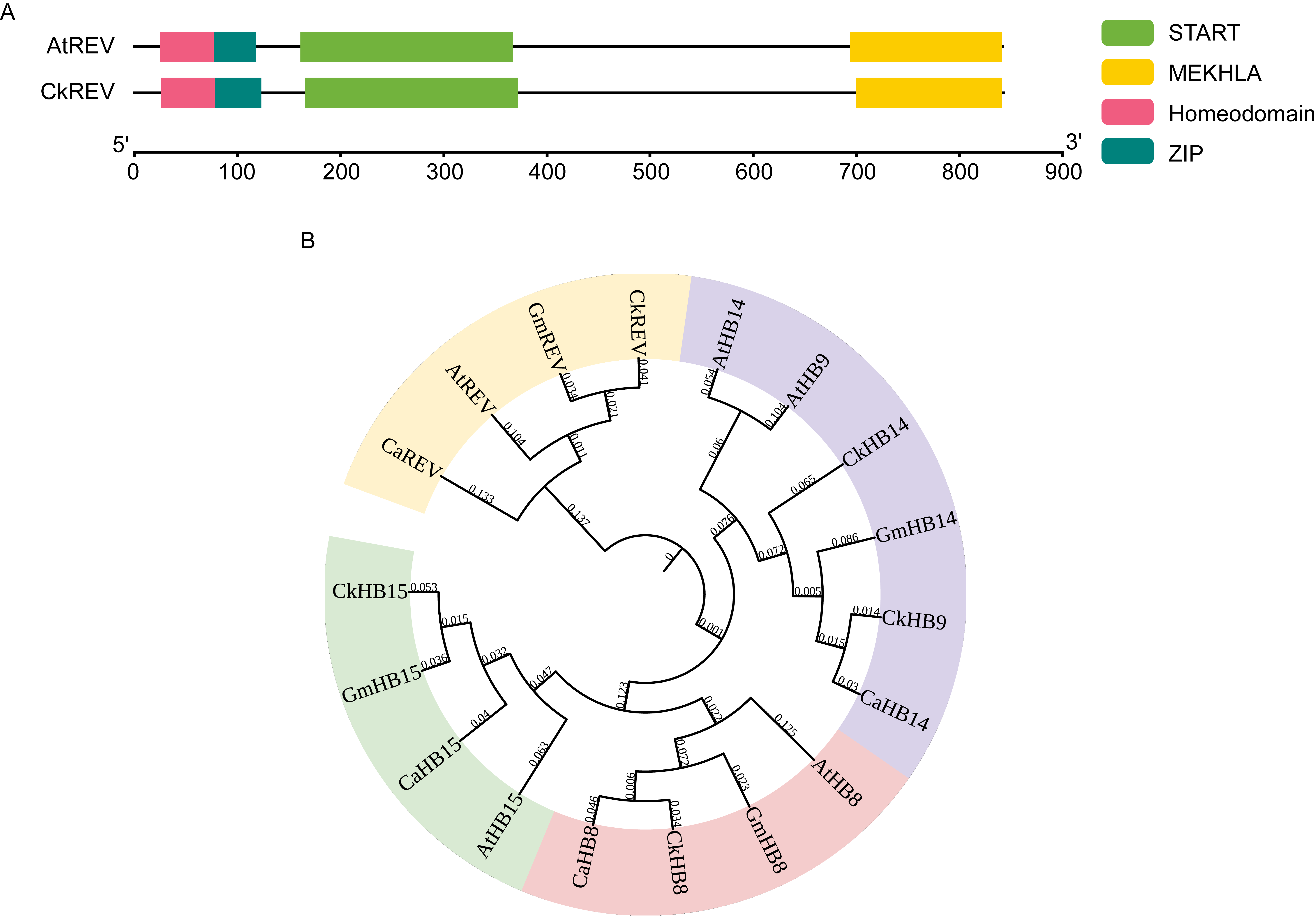
Supplementary Figure 1.** Phylogenetic analysis and conserved domain analysis of *C. korshinskii* HD-ZIP III family TF. (A) Conserved domain analysis of CkREV and AtREV. (B) Phylogenetic analysis of HD-ZIP III family of *C. korshinskii*, *A. thaliana*, *Glycine max* and *Cicer arietinum.* Bootstrap support(1 000 repetitions) is shown for each node.

***
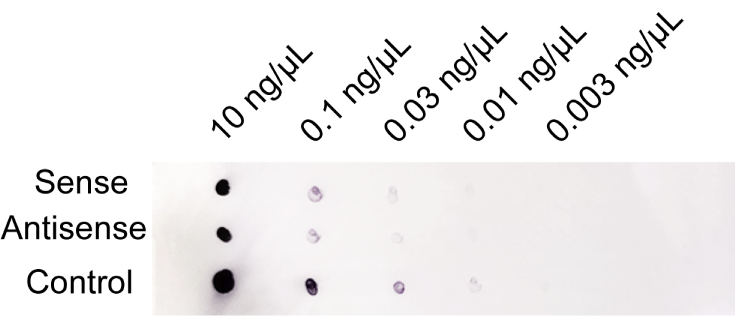
*Supplementary Figure 2.** *CkREV* in situ hybridization probe labeling efficiency spot detection.

**
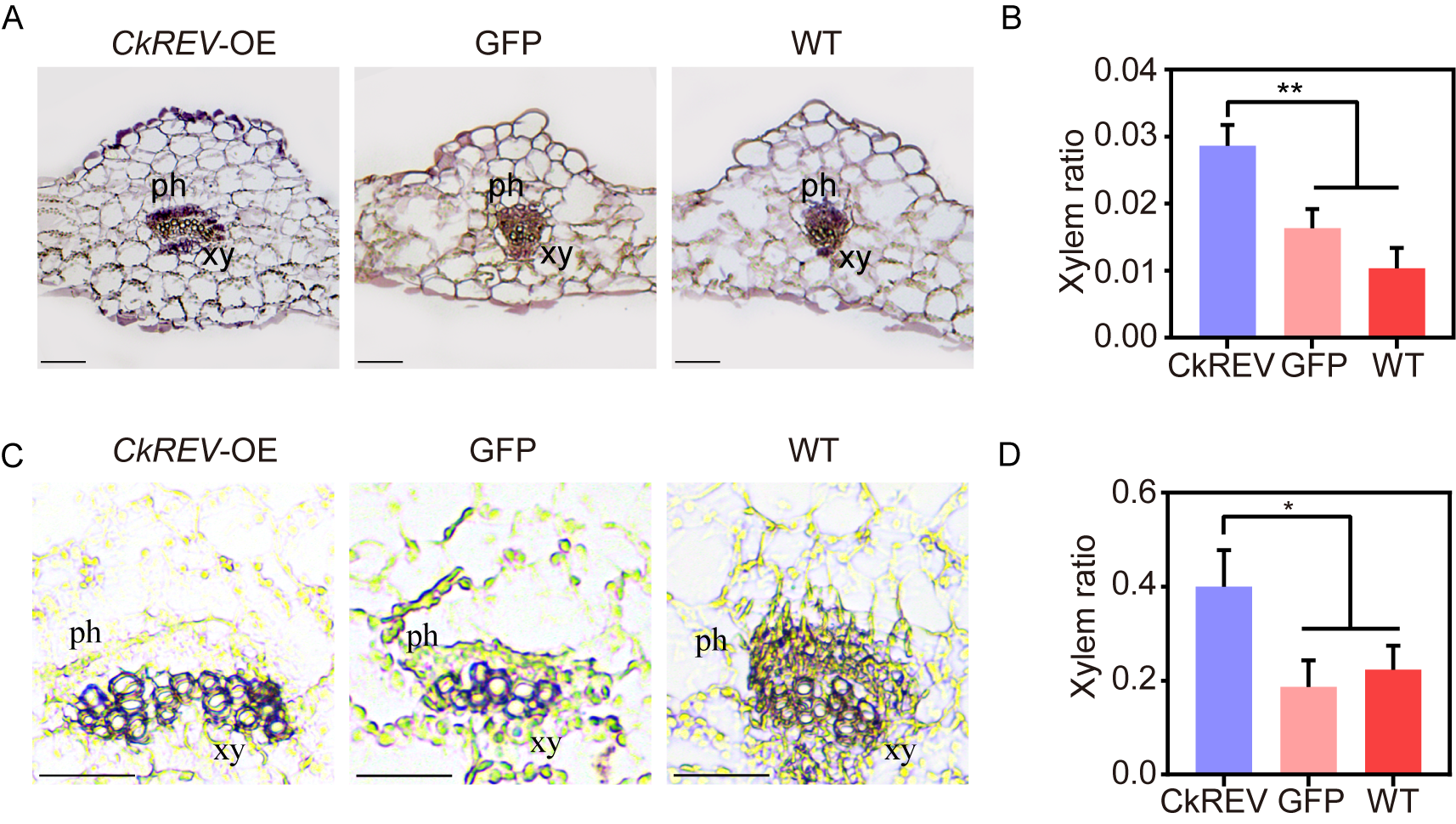
Supplementary Figure 3.** Cellulose and lignin staining of transgenic *A. thaliana* leaf vein sections. (A) Zinc iodide chloride staining on leaf vein sections of *A.thaliana* *CkREV*-OE lines. (B) The ratio of stained xylem region to the entire vascular bundle. (C) Phloroglucinol staining on leaf vein sections of *A.thaliana* *CkREV*-OE lines. (D) The ratio of stained xylem region to the entire vascular bundle. ph, phloem, xy, xylem.

**
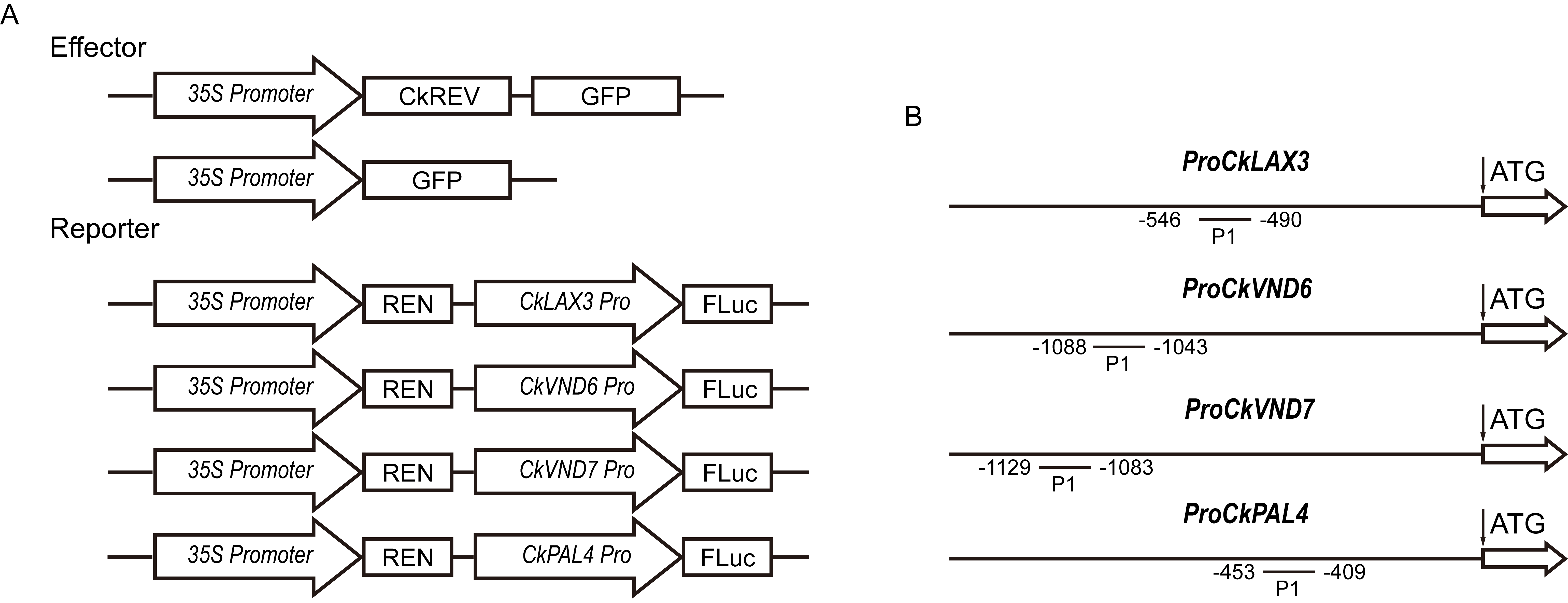
Supplementary Figure 4.** Pattern graph of DUAL-LUC assay on the regulation of CkREV on *CkLAX3*, *CkVND6*, *CkVND7* and *CkPAL4.*

**
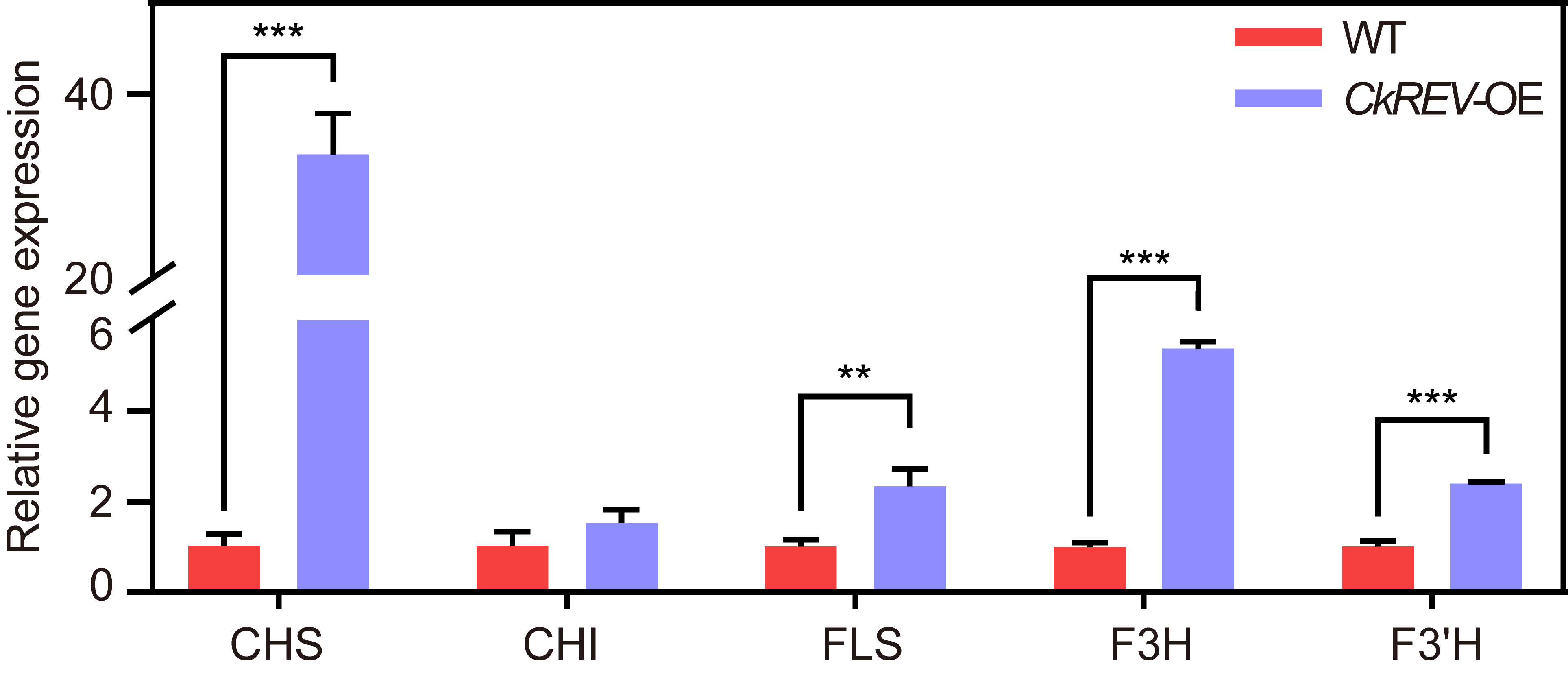
Supplementary Figure 5.** Expression levels of flavonoid biosynthesis-related genes in *A.thaliana* *CkREV*-OE line. Error bars indicate SD (n = 3). Student’s t test is employed to measure statistical significance between two samples with confidence level at 0.95 (**, *P* <0.05; ***, *P* <0.001).

| **Primers** | **Primer sequence（5'→3'）** |
| --- | --- |
| RT-CkREV-F | ATTCAAAACCAGGCGATAA |
| RT-CkREV-R | GAAATCACGCTGCGGACA |
| RT-Ckβ-actin F | GGCTGTCCTCTCCCTCTATGC |
| RT-Ckβ-actin R | CGAACAATTTCCCGCTCAG |
| RT-CkLAX2-F | TAGTTTCCACCTTCAATGAGACA |
| RT-CkLAX2-R | CAAATAGAACCACCATGCCAGAG |
| RT-CkLAX3-F | AGGCTCTTCTGGCATGGTG |
| RT-CkLAX3-R | CCCATCAGTCCATAAAAAAGC |
| RT-CkVND6-F | AAGAAGGCTGGGTTGTATGC |
| RT-CkVND6-F | GTTGGAGAAAAGCATCGTGGT |
| RT-CkVND7-F | TTGGAAGGAGCAGAAGATTGAA |
| RT-CkVND7-F | GAAGAAGGATGGGTTGTGTGTAG |
| RT-CkCesA7-F | GTGGCACGGCTTGTTATTC |
| RT-CkCesA7-R | TGATCGAAAGGCGGTCA |
| RT-CkCesA8-F | TGATGGTATTGATCGAAGTGAT |
| RT-CkCesA8-R | GCAGGATGAAGATTTTGGTAA |
| RT-CkIRX9-F | AGATGGGTTCAGTAGAAAGGTC |
| RT-CkIRX9-R | TCTTTGGCAAGGAGGTCAC |
| RT-CkPAL4-F | TACTTTGCCACACAATGCAAC |
| RT-CkPAL4-R | ATGTTGTGATTGAGTAGTTTTGC |
| RT-CkPAL2-F | TGGATTATGGTTTCAAGGGAGC |
| RT-CkPAL2-R | ACATGGGTTGTGACCGGATT |
| RT-CkCAD1-F | ATGGGTAGCCTTGAATCGGA |
| RT-CkCAD1-R | GCCCTGTGTTTCTCAGCGTGTA |
| RT-CkAS1-F | GGTTAGGAAAGTGGTGGGAAG |
| RT-CkAS1-R | GCAATGCTGGTGCGGGG |

**Supplementary Table 1.** *C. korshinskii* qRT-PCR primers used in this study.

| **Primers** | **Primer sequence（5'→3'）** |
| --- | --- |
| RT-Atβ-actin2-F | TTACCCGATGGGCAAGTC |
| RT-Atβ-actin2-R | GCTCATACGGTCAGCGATAC |
| RT-AtVND6-F | GATCATATGATCATGATGAACACAAGT |
| RT-AtVND6-F | CGTGTGTGTATTTTGAGCCCAAGAGTAGAA |
| RT-AtVND7-F | TAGCTTCCAAAAAGACACGCTTATG |
| RT-AtVND7-F | GTGGCATTGACGATTGCATTATATT |
| RT-AtLAX2-F | TTCATTGTTGGATTCGGGTTCG |
| RT-AtLAX2-R | AGGATGGCTGATTGGAGGAGGT |
| RT-AtLAX3-F | GGTATTCGTAGTTGGGTTCGGG |
| RT-AtLAX3-R | GAGGGCATTGGTAGCATTTGGT |
| RT-AtCesA8-F | CAATTTGGAAGAACAGAGTGGAG |
| RT-AtCesA8-R | CCATGTGCTGTTGGGTAGGA |
| RT-AtIRX9-F | TCCACAAAAATAAAAACGGAACTCA |
| RT-AtIRX9-R | GAAGCCAGTGAAGAAACCCATTACA |
| RT-AtCHS-F | CGCATCACCAACAGTGAACAC |
| RT-AtCHS-R | TCCTCCGTCAGATGCATGTG |
| RT-AtCHI-F | CCGGTTCATCGATCCTCTTC |
| RT-AtCHI-R | ATCCCGGTTTCAGGGATACTATC |
| RT-AtFLS-F | CCGTCGTCGATCTAAGCGAT |
| RT-AtFLS-R | CGTCGGAATCCCGTGGT |
| RT-AtF3H-F | GGAAGGTTGGGTGAAAGTGA |
| RT-AtF3H-R | TGTAGCAGCAAGGTAATGGTT |
| RT-AtWRKY53-F | CAGAGTCAAACCAGCCATTACCC |
| RT-AtWRKY53-R | CGTCTTTACCATCATCAAGCCCA |

**Supplementary Table 2.** *A. thaliana* qRT-PCR primers used in this study.

| **Primers** | **Primer sequence（5'→3'）** |
| --- | --- |
| LAD1 | ACGATGGACTCCAGAGCGGCCGCVNVNNNGGAA |
| LAD2 | ACGATGGACTCCAGAGCGGCCGCBNBNNNGGTT |
| LAD3 | ACGATGGACTCCAGAGCGGCCGCVVNVNNNCCAA |
| LAD4 | ACGATGGACTCCAGAGCGGCCGCBDNBNNNCGGT |
| AC1 | ACGATGGACTCCAGAG |
| CkVND7-1 | TGCTCACTCACTTTACGCATTG |
| CkVND7-2 | ACGATGGACTCCAGTCCGGCCCTAAGGTTTTCCTCATCCC |
| CkVND7-3 | TGTTGCTTTCCAAAATCCAG |
| CkVND6-1 | TCATTCGCTTCTTGAACACC |
| CkVND6-2 | ACGATGGACTCCAGTCCGGCCTGGGGCTCGTCCTTT |
| CkVND6-3 | TTCCAGAATCCTGCTTTTGTAG |
| CkLAX3-1 | CAGTGTTTGCCTAAAAGTCCATC |
| CkLAX3-2 | GCTTCCCATCAGTCCATAAAA |
| CkLAX3-3 | CCACCATGCCAGAAGAGC |
| CkPAL4-1 | CCTTCCTGTAAGGAGTCCTGCT |
| CkPAL4-2 | TGGTGTTATGTTGTGATTGAGTAGT |
| CkPAL4-3 | CCTTGAAGGAGGGTATTGATT |

**Supplementary Table 3.** *C. korshinskii* genome walking primers used in this study.
